# Clinical and biochemical efficacy zoledronic acid and denosumab combination: focus serum inflammatory factor level (serum ifcs), bone gla protein (bgp), and bone turnover markers b-collagen degradation product (b-ctx), and procollagen type 1 n-terminal propeptide (p1np)

**DOI:** 10.5937/jomb0-51444

**Published:** 2025-06-13

**Authors:** Lingyan Kong, Jun Ma

**Affiliations:** 1 Yuyao People's Hospital, Department of Endocrinology, Yuyao, China

**Keywords:** zoledronic acid, denosumab, postmenopausal osteoporosis, serum inflammatory cytokines, zoledronska kiselina, denosumab, postmenopauzalna osteoporoza, serumski inflamatorni citokini

## Abstract

**Background:**

Postmenopausal osteoporosis (PMOP) is a prevalent metabolic bone disorder characterized by decreased bone mineral density (BMD) and skeletal fragility, leading to increased susceptibility to fractures. The therapeutic efficacy of zoledronic acid and denosumab, two widely used agents in the treatment of osteoporosis, was investigated in this study. The primary objective was to evaluate the clinical effects of zoledronic acid and denosumab on serum inflammatory cytokine (IFC) levels and BMD in PMOP patients.

**Methods:**

A prospective, non-blinded, randomized controlled trial was conducted at our hospital from March 2021 to March 2024. Eighty PMOP patients were recruited and randomly assigned to either a control group (CG, n=40) or a treatment group (TG, n=40). The CG received zoledronic acid plus traditional treatment, while the TG received zoledronic acid plus denosumab plus traditional treatment. Clinical symptom improvement and changes in BMD were assessed and compared between the two groups. Serum IFC levels, including bone Gla protein (BGP) and bone turnover markers b-collagen degradation product (b-CTX) and procollagen type 1 N-terminal propeptide (P1NP), were measured.

**Results:**

Compared to the CG, patients in the TG demonstrated significantly increased BMD (P<0.05) and decreased levels of serum IFCs, BGP, and bone turnover markers (P<0.05). Additionally, the incidence of adverse reactions was significantly lower (P<0.05) in the TG, and the total effective rate of clinical treatment was significantly higher (P<0.05).

**Conclusions:**

The combination of zoledronic acid and denosumab exhibited improved clinical efficacy in PMOP patients, as evidenced by enhanced BMD and reduced serum IFC levels. These findings suggest that this combined treatment regimen may promote the treatment of osteoporosis by suppressing inflammatory responses, thereby providing a novel therapeutic approach for the management of PMOP.

## Abstract (sr)

**Uvod:**

Postmenopauzalna osteoporoza (PMOP) je prevalentan metabolički poremećaj kostiju, koji karakteriše smanjena gustina minerala u kostima (BMD) i krhkost skeleta, što dovodi do povećane podložnosti frakturama. Ova studija je ispitala terapijsku efikasnost zolendronske kiseline i denosumaba, dva široko korišćena sredstva u lečenju osteoporoze. Glavni cilj bio je da se ocene klinički efekti zolendronske kiseline i denosumaba na nivoe serumskih inflamatornih citokina (IFC) i BMD kod pacijenata sa PMOP.

**Metode:**

Sprovedena je prospektivna, "neslepa", randomizovana kontrolisana studija u našoj bolnici od marta 2021. do marta 2024. godine. Izabrano je osamdeset pacijenata sa PMOP-om i nasumično podeljeno u kontrolnu grupu (CG, n=40) ili grupu za lečenje (TG, n=40). CG je primila zolendronsku kiselinu uz tradicionalni tretman, dok je TG primila zolendronsku kiselinu plus denosumab plus tradicionalni tretman. Poboljšanje kliničkih simptoma i promena u BMD-u su procenjivani i upoređivani između dve grupe. Mereni su nivoi serumskih inflamatornih citokina (IFC), uključujući koštani Gla protein (BGP) i markere metabolizma kostiju, proizvod degradacije b-kolagena (b-CTX) i prokolagen tipa 1 N-terminalni propeptid (P1NP).

**Rezultati:**

U pore|enju sa CG, pacijenti u TG su pokazali značajno povećanje BMD-a (P<0,05) i smanjenje nivoa serumskih IFC, BGP i markera metabolizma kostiju (P<0,05). Dodatno, incidenca neželjenih reakcija je bila značajno niža (P<0,05) u TG, dok je ukupan efekat kliničkog lečenja bio značajno viši (P<0,05).

**Zaključak:**

Kombinacija zolendronske kiseline i denosumaba je pokazala poboljšanu kliničku efikasnost kod pacijenata sa PMOP, što je dokazano poboljšanjem BMD i smanjenjem nivoa serumskih IFC. Ovi nalazi sugerišu da ovaj kombinovani režim lečenja može doprineti lečenju osteoporoze potiskivanjem inflamatornih odgovora, čime se pruža novi terapijski pristup za lečenje PMOP.

## Introduction

Postmenopausal osteoporosis (PMOP) is a common metabolic bone disorder with decreased bone mineral density (BMD) and skeletal fragility, leading to fractures and other severe complications. This condition arises from estrogen deficiency in women after menopause, where bone resorption exceeds bone formation, resulting in insufficient bone mass and increased susceptibility to fragility fractures, significantly impacting patients’ quality of life [1]. With the global ageing population trend intensifying, the incidence of PMOP continues to rise, making skeletal health a prominent concern in the field of public health. Studies indicate that postmenopausal women over 50 are more than six times as likely to develop PMOP compared to age-matched men, with risk increasing with age [2]. Among elderly individuals, osteoporotic hip fractures are particularly devastating, with 21%–30% of patients succumbing within one year [3]. Consequently, early prevention and treatment of osteoporosis are imperative to promote skeletal health and have become a critical objective for global healthcare systems.

Currently, the diagnosis of PMOP primarily relies on BMD measurements, which are considered the gold standard [4]. Nevertheless, due to its slow-changing nature, BMD has certain limitations in confirming diagnosis. Clinical treatment approaches for PMOP mainly involve supplementation with calcium and vitamin D, using bone resorption inhibitors (such as bisphosphonates), and using bone-forming agents. Zoledronic acid is clinically utilized to treat skeletal disorders by inhibiting osteoclast proliferation, reducing bone resorption, and lowering fracture risk [5]. A meta-analysis study demonstrated that with bisphosphonate treatment, patients with an absolute risk of 0.010 needed 12.4 months of treatment to avoid fracture occurrence, whereas those with an absolute risk of 0.005 required 20.3 months of treatment to prevent hip fractures, suggesting that bisphosphonate treatment is most beneficial for PMOP women with an expected lifespan exceeding 12.4 months [6]. Nevertheless, using bisphosphonates alone for PMOP treatment requires prolonged treatment cycles and presents significant limitations, especially in severe cases. Denosumab, a humanized monoclonal antibody, exerts highly selective inhibition of bone resorption without affecting bone-forming protein activity. Wang et al. [7] found that denosumab and teriparatide exhibited better efficacy in improving spinal BMD relative to ibandronate. Denosumab demonstrated superior effects in improving radius BMD and radius density compared to teriparatide, with additional benefits in preventing vertebral fractures and a lower incidence of adverse reactions (ARs) compared to zoledronic acid [7].

Hence, denosumab is considered a potentially effective treatment for PMOP. Studies have shown that with long-term use of bisphosphonates alone, the incidence of ARs, such as oral intolerance, dementia, and poor absorption, increases [8]. Recent research has conducted bone morphogenetic protein (BMP) screening in PMOP patients, measuring serum levels interleukin-6 (IL-6), IGF-1, BMP-2, VEGF, leptin, and FGF23. The results indicated that IGF-1 and leptin are key PMOP biomarkers [9]. Furthermore, an *in vivo* study revealed changes in serum concentrations of IL-6, malondialdehyde, nitrate, alkaline phospha tase, and phosphatein PMOP rats [10]. Hence, PMOP may be associated with changes in patients’ serum inflammatory cytokine (IFC) levels. Inves tigating these changes in serum IFCs is crucial for a deeper understanding of disease mechanisms and identifying new treatment strategies.

Currently, zoledronic acid and denosumab are widely utilized in osteoporosis therapy; however, their clinical efficacy and impact on serum IFC levels remain incompletely understood. Moreover, there is limited research on whether the combination of zoledronic acid and denosumab reduces the incidence of ARs. This work aimed to demonstrate the clinical efficacy of combined zoledronic acid and denosumab therapy in PMOP patients and investigate its influence on serum IFC levels. This research seeks to provide new evidence and theoretical foundations to enhance the therapeutic outcomes of PMOP therapy.

## Materials and methods

This clinical trial study was conducted on 80 PMOP patients who visited Yuyao People’s Hospital from March 2021 to March 2024. Yuyao People’s Hospital institutional review board confirmed the study’s protocol with reference code #35714837A20. Patients were randomly assigned into a treatment group (TG, n=40) and a control group (CG, n=40).

Inclusion criteria: patients diagnosed with PMOP regarding the diagnostic gold standard of the *UK clinical guideline for the prevention and treatment of osteoporosis (2021)* [11], confirmed by X-ray ex amination; menopausal duration ≥2 years; cyclophosphamide (CTX) ≥0.45 ng/mL, with no notable increase or decrease in levels of procollagen I N-terminal propeptide (PINP).

Exclusion criteria: patients with bone metabolism-related diseases; severe renal or hepatic dysfunction; malignancies; contraindications to the study medications; patients who had taken prohibited medications (excluding calcium supplements) before enrollment.

### Randomization

Patients were randomly assigned to either the Treatment Group (TG) or Control Group (CG) using a computer-generated randomization sequence. The randomization ratio was 1:1, with 40 patients in each group. To ensure balance between groups, patients were stratified by age (<60, ≥60) and menopausal duration (<5, ≥5 years). The randomization se quence was concealed from the researchers and patients until the allocation was revealed, minimizing selection bias. This study did not employ blinding, meaning that both patients and researchers were aware of the treatment assignments. Patients knew which treatment they were receiving, and researchers knew which treatment they were administering.

### Interventions

The CG was treated with zoledronic acid (Zoledronic Acid Injection, CHIATAI TIANQING PHARMACEUTICAL GROUP CO.LTD., China; specification: 100 mL: 5 mg), administered intravenously at 5 mg per dose, once per year, with infusion controlled to complete over 15 minutes, preceded, and followed by infusion of 250 mL of 0.9% saline solution. The TG received additional denosumab (Deno sumab injection, manufactured by Amgen Manu facturing Limited (AML); specification: 60 mg (1.0 mL) per vial), administered subcutaneously in the abdomen at 60 mg per dose, every 6 months, in addition to the treatment protocol of CG. Both groups received calcium supplementation and vitamin D3 (manufactured by Zhejiang Xinhe Pharmaceutical Co., Ltd., China; specification: 1 mL: 7.5 mg) through out the 12-month treatment period.

### Outcome measures

### Serum IFC analysis

On the second day after patient enrollment and the morning following treatment, 10 mL of venous blood was drawn and centrifuged at 1,500 r/15 min to collect the supernatant, which was then stored frozen. Serum inflammatory cytokines (IL-1α, IL-6, TNF-α, IL-1β, and IFN-γ) were measured using ELISA kits (Human Inflammatory Cytokine ELISA Kit, YAB-IFC-10, Shanghai Yuanye Bio-Technology Co., Ltd.) following the manufacturer’s instructions. Briefly, 10 mL of venous blood was drawn and centrifuged at 1,500r/15 min to collect the supernatant, which was then stored frozen. On the analysis day, the samples were thawed and diluted 1:10 with sample diluent, then added to microtiter plates pre-coated with monoclonal antibodies specific for each cytokine. The plates were incubated at 37°C for 2 hours, followed by the addition of biotinylated detection antibodies and streptavidin-HRP conjugate. The reaction was developed with TMB substrate solution and stopped with stop solution, and the absorbance was read at 450 nm using a microplate reader. The concentrations of each cytokine were calculated based on the standard curves generated using the provided standard solutions, with sensitivities of 1 pg/mL (IL-1α), 2 pg/mL (IL-6), 5 pg/mL (TNF-α), 1 pg/mL (IL-1β), and 5 pg/mL (IFN-γ).

The study observed changes in BMD levels, bone turnover markers, bone pain, and incidence of ARs in both groups pre- and post-treatment.

(1) BMD was tested as follows: BMD at the lumbar spine and femoral neck was measured using the EXA-PRESTO dual-energy X-ray absorptiometry system (manufactured by Osteosys Co., Ltd., South Korea). A T-score was utilized as the evaluation criterion for this work.


(1) T=\frac{D_{Bone}-D_{Bone}}{S_{D_{Bone}}}

Note: *D* represents density; is the mean BMD of normal young adults; is the standard deviation of BMD in normal young adults.

(2) Bone pain assessment was as follows: patient’s bone pain was assessed using the visual analogue scale (VAS), with a maximum score of 10.

(3) Bone Glaprotein (BGP) level was tested as follows: serum BGP levels were measured using a human osteocalcin (OC/BGP) ELISA kit (manufactured by Sangon Biotechnologies, Inc., China), following the same methodology as described in section the section Outcome measures.

(4) ARs such as nausea, dyspepsia, dizziness, hypertension, and joint pain during medication were observed.

(5) Bone turnover marker analysis as follows: serum levels of bone turnover markers, including β-collagen degradation product (β-CTX) and P1NP, were measured using electrochemiluminescence immunoassay.

Clinical efficacy was assessed as follows: marked effectiveness: no pain sensation and an increase in BMD ≥25%; effective: reduction in pain sensation with no decrease in BMD upon testing; ineffective: no notable enhancement in pain sensation or BMD level or worsening of symptoms.


(2) \eta =\frac{N_{Clearly effective}+N_{Effective}}{N_{ALL}}

Note: η represents treatment efficiency; *N* represents the number of patients.

### Statistical analysis

This work processed the research data using SPSS version 27.0 statistical software. Descriptive statistics for continuous data, denoted as mean ± standard deviation (±s), were compared employing independent samples t-test. Categorical data denoted as percentages [n (%)] were compared employing the chi-square (χ^2^) test. The significance level was set at *P*<0.05.

## Results

**Figure 1:**
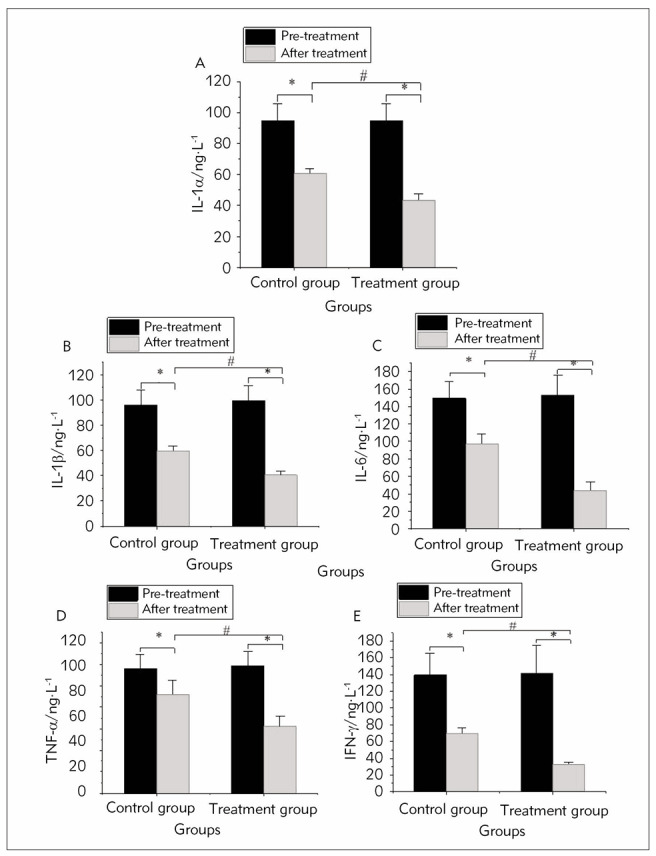
Changes in serum IFC levels pre- and post-treatment in two groups.<br>Note: *P<0.05 vs. pre-treatment levels; #P<0.05 vs. CG.

The treatment group (TG) and control group (CG) exhibited comparable demographic characteristics at baseline, with mean ages of 66.25±10.15 years and 64.19±9.96 years, respectively (p > 0.05). Menopausal duration ranged from 4 to 23 years in the TG and 4 to 22 years in the CG, while mean body weight was 59.15±9.74 kg and 58.94±10.22 kg, respectively (p>0.05). These similarities in baseline characteristics ensured the validity of the study’s comparative design. Table 1 Figure 1

**Table 1:** Baseline and outcome characteristics of study subjects.

Characteristic	Treatment Group (TG)	Control Group (CG)	p-value
Age (years)	66.25±10.15	64.19±9.96	0.235
Weight (kg)	59.15±9.74	58.94±10.22	0.811
Lumbar Spine BMD			
Pre-treatment	0.85±0.12	0.83±0.11	0.562
Post-treatment	1.02±0.14	0.92±0.13	0.012
Femoral Neck BMD			
Pre-treatment	0.78±0.10	0.76±0.11	0.454
Post-treatment	0.95±0.12	0.85±0.13	0.028
Bone Pain Scores			
Pre-treatment	7.5±1.5	7.2±1.4	0.523
Post-treatment	3.5±1.2	5.2±1.5	0.008
Serum Osteocalcin (ng/mL)			
Pre-treatment	12.5±2.5	12.2±2.8	0.725
Post-treatment	8.5±1.8	10.5±2.2	0.042
Serum β-CTX (ng/mL)			
Pre-treatment	0.95±0.2	0.92±0.2	0.623
Post-treatment	0.55±0.15	0.72±0.18	0.015
Serum P1NP (ng/mL)			
Pre-treatment	45.2±10.5 ng/mL	42.1±10.2 ng/mL	0.382
Post-treatment	32.5±8.2 ng/mL	39.2±9.5 ng/mL	0.031
Bone Pain Scores (VAS)			
Pre-treatment	7.2±1.5	7.0±1.4	0.567
Post-treatment	2.5±1.2	4.2±1.5	0.005

Following 12 months of intervention, significant improvements in bone mineral density (BMD) were observed in both groups (p<0.05). However, the magnitude of the BMD increase was significantly greater in the TG compared to the CG (p<0.05). Pretreatment BMD values at the lumbar spine and femoral neck were comparable between groups (p>0.05). In contrast, post-treatment BMD levels demonstrated a notable increase in both groups, with the TG exhibiting a more pronounced effect.

Serum osteocalcin levels were analyzed at baseline and post-treatment. Although pre-treatment levels were comparable between groups (p>0.05), a significant decrease was observed in both groups following 12 months of treatment (p<0.05). Notably, the reduction in serum osteocalcin levels was more pronounced in the TG compared to the CG (p<0.05), indicating a significant between-group difference.

Adverse reactions (ARs) were also assessed, revealing a significantly lower incidence in the TG compared to the CG (15.0% vs. 47.5%, p<0.05). Specifically, the TG experienced fewer cases of nausea, fever, gastrointestinal discomfort, dizziness, elevated blood pressure, and joint pain than the CG.

The efficacy of the treatment was evaluated by the number of patients achieving effective treatment outcomes. The TG demonstrated a significantly higher proportion of responders (97.5%) compared to the CG (80.0%, p<0.05), indicating a substantial difference in treatment efficacy.

After 12 months of treatment, the levels of serum IFCs TNF-α, IL-1α, IL-6, IL-1β, and IFN-γ markedly decreased in both groups (*P*<0.05). Additionally, the reduction in serum IFC levels was more pronounced in TG versus CG (*P*<0.05), indicating a substantial difference (*Figure 3*).

## Discussion

Fragility fractures are common in patients with PMOP, characterized by a gradual decline in BMD due to decreased estrogen levels following meno pause. PMOP patients often present clinically with back pain and leg pain, and some may experience height loss, with severe cases exhibiting kyphosis. Among postmenopausal women over 65, 50% experience mobility limitations, significantly impacting their daily lives [12]. Our work demonstrated that the efficacy of zoledronic acid combined with denosumab in treating PMOP was markedly superior to that of using zoledronic acid alone, indicating a notable effect of the combination therapy.

### Comparison with existing literature

Zoledronic acid works by binding to calcium ions in bone, inhibiting the activity of bone-resorbing cells, disrupting bone turnover, and promoting osteoblast activity, which helps maintain BMD and reduces fracture risk [13]. Denosumab specifically targets and inhibits the RANKL, thereby blocking its binding to its receptor RANK [14]. This action reduces the formation and activity of osteoclasts, inhibiting bone resorption. Additionally, denosumab significantly increases BMD, thus improving the imbalance in bone resorption. Combining medications reveals significantly higher BMD in TG versus CG, demonstrating a notable effect of the treatment in increasing BMD. In a Mate analysis study, it was proposed that denosumab and zoledronic acid are safe for treating fragility fractures in non-cancerous adults aged 50 years and older with osteoporosis, with ARs within manageable ranges [15]. Zoledronic acid promotes bone mass increase and inhibits bone resorption, while denosumab activates osteoblast activity and promotes bone formation. The combined application of zoledronic acid and denosumab significantly increases BMD levels. This work also found that the bone VAS scores in TG were drastically inferior to those in CG, indicating that the treatment effectively alleviates bone pain with more pronounced therapeutic effects. Osteoporotic pain is likely alleviated due to the promotion of BMD and mass by zoledronic acid and denosumab, which mitigate factors like bone loss and inflammatory responses [16]. Crack et al. [17] demonstrated that zoledronic acid can mitigate the loss of proximal femoral strength and promote the reconstruction of trabecular bone. Other research has found that zoledronic acid can reduce bone pain caused by osteodystrophy and lower bone turnover marker levels [18]. This aligns with the findings from our study. PMOP-induced bone pain may be related to inflammatory responses. Our study found notably elevated serum IFCs IL-1α, IL-1β, IL-6, TNF-α, and IFN-γ levels in PMOP patients, indicating an inflammatory response within the patients. Post-treatment, the synthesis of IFCs in TG was inhibited, indicating that zoledronic acid and denosumab therapy effectively reduced inflammation levels. Analysis suggested that the reduction in IFCs IL-1α, IL-1β, IL-6, TNF-α, and IFN-γ may be associated with the reduction in patient bone pain. Currently, β-CTX and PINP are markers of bone formation in clinical practice. The TG exhibited a more pronounced decrease in serum osteocalcin levels, β-CTX, and PINP levels, indicating that bisphosphonates and denosumab therapy can reduce levels of bone turnover markers. Zoledronic acid inhibits osteoclast activity and reduces osteoblast activity, thereby modulating bone turnover. The combination use of denosumab further enhances this effect. These findings are in line with the results of Hu et al. [19]. Further more, combining zoledronic acid with other treatments, such as percutaneous vertebroplasty, has been shown to improve bone mineral density and reduce pain in elderly patients with osteoporotic lumbar vertebral compression fractures [20].

### Implications for clinical practice

Zoledronic acid and denosumab both exhibit certain ARs, such as nausea, dyspepsia, dizziness, hypertension, and joint pain during the treatment of PMOP. When utilized in combination, the incidence of ARs decreases, indicating that combination therapy has a safety advantage. These ARs typically diminish over time. Hence, post-treatment, patients should be advised to drink plenty of water, engage in appropriate outdoor activities, and get sufficient sunlight exposure to enhance calcium absorption. In summary, the efficacy of zoledronic acid and denosumab in treating PMOP is notable, as they can improve cure rates, suppress levels of IFCs and bone turnover markers, reduce bone pain associated with osteoporosis, and inhibit osteoporotic progression. This combination represents an effective therapeutic option.

### Limitations of the study and future research directions

This study has several limitations that should be acknowledged. Firstly, the lack of blinding may have introduced bias in the results, and future studies should consider using double-blinding to minimize bias. Additionally, the small sample size of 80 patients may not be representative of the larger population, and future studies should aim to recruit a larger sample size to increase the generalizability of the results. Furthermore, the short follow-up period of 12 months may not be sufficient to capture the long-term effects of the treatment, and future studies should consider a longer follow-up period to assess the sustained efficacy and safety of the treatment.

Future research directions should address these limitations and explore new avenues of investigation. Long-term follow-up studies should be conducted to assess the sustained efficacy and safety of the combined treatment of zoledronic acid and denosumab. Comparative studies should also be conducted to compare the efficacy and safety of this treatment regimen with other treatment regimens, such as teriparatide or romosozumab. Additionally, mechanistic studies should be conducted to investigate the mechanisms by which the combined treatment of zoledronic acid and denosumab exerts its effects on serum IFC levels and BMD. Finally, cost-effectiveness analyses should be conducted to assess the economic implications of this treatment regimen compared to other treatment regimens.

## Conclusion

In this work, the combination therapy of zoledronic acid and denosumab for PMOP demonstrated excellent clinical efficacy with fewer ARs. It notably improved BMD, reduced fracture risk, and lowered levels of IFCs and bone turnover markers to some extent. Moreover, its therapeutic effects were long-lasting, facilitating management and exhibiting good tolerability and safety. Future research should investigate the long-term efficacy and safety of this combination therapy to provide more effective strategies for clinical treatment.

## Dodatak

### Conflict of interest statement

All the authors declare that they have no conflict of interest in this work.
